# A remote hypertension management program clinical algorithm

**DOI:** 10.1002/clc.23919

**Published:** 2022-09-24

**Authors:** Hunter Nichols, Christopher P. Cannon, Benjamin M. Scirica, Naomi D. L. Fisher

**Affiliations:** ^1^ Division of Cardiovascular Medicine Boston Massachusetts USA; ^2^ Department of Pharmacy Services Boston Massachusetts USA; ^3^ Brigham and Women's Hospital Boston Massachusetts USA; ^4^ Harvard Medical School Boston Massachusetts USA; ^5^ Division of Endocrinology Diabetes and Hypertension Boston Massachusetts USA

**Keywords:** algorithmic management, Angiotensin II receptor blockers (ARBs) and Angiotensin‐converting enzyme (ACE) inhibitors, beta blockers, calcium channel blockers (CCBs), CDTM, collaborative drug therapy management, hypertension, mineralocorticoid receptor antagonists, pharmacy, thiazide diuretics

## Abstract

**Introduction:**

Hypertension is the leading risk factor for death, affecting over one billion people worldwide, yet control rates are poor and stagnant. We developed a remote hypertension management program that leverages digitally transmitted home blood pressure (BP) measurements, algorithmic care pathways, and patient–navigator communications to aid patients in achieving guideline‐directed BP goals.

**Methods:**

Patients with uncontrolled hypertension are identified through provider referrals and electronic health record screening aided by population health managers within the Mass General Brigham (MGB) health system. Non‐licensed patient navigators supervised by pharmacists, nurse practitioners, and physicians engage and educate patients. Patients receive cellular or Bluetooth‐enabled BP devices with which they monitor and transmit scheduled home BP readings. Evidence‐based medication changes are made according to a custom hypertension algorithm approved within a collaborative drug therapy management (CDTM) agreement with MGB and implemented by pharmacists.

Using patient‐specific characteristics, we developed different pathways to optimize medication regimens. The renin–angiotensin–aldosterone system‐blocker pathway prescribed ARBs/ACE inhibitors first for patients with diabetes, impaired renal function, and microalbuminuria; the standard pathway started patients on calcium channel blockers. Regimens were escalated frequently, adding thiazide‐type diuretics, and including beta blockers and mineralocorticoid receptor antagonists if needed.

**Discussion:**

We have developed an algorithmic approach for the remote management of hypertension with demonstrated success. A focus on algorithmic decision‐making streamlines tasks and responsibilities, easing the potential for scalability of this model. As the backbone of our remote management program, this clinical algorithm can improve BP control and innovate the management of hypertension in large populations.

## 1 INTRODUCTION

Hypertension is the leading risk factor for death, affecting over one billion people worldwide, yet control rates are poor and stagnant.^1^, ^2^, ^3^ We developed a remote hypertension management program that leverages digitally transmitted scheduled home blood pressure (BP) measurements, algorithmic care pathways, and patient–navigator communications. Pharmacists prescribe medications to aid patients in achieving guideline‐directed BP goals.^2^, ^4^ Evidence‐based medication initiations, titrations, and discontinuations are implemented using an established drug‐treatment algorithm approved within a collaborative drug therapy management (CDTM) agreement with Mass General Brigham (MGB). State legislation provides CDTM agreements as a mechanism for pharmacists to prescribe under care algorithms designed with physicians.^5^ Programs with pharmacists working under a CDTM agreement have demonstrated positive impacts on patient care and helped reduce physicians' workloads by providing care via telemedicine and other digital communication avenues.^6^, ^7^

Chronic disease states like hypertension are particularly well‐suited for remote management and thus CDTM‐guided care.^7^ Further, telehealth and remote management strategies became vital during the COVID‐19 pandemic and are likely here to stay.^8^ We have previously reported on the clinical success of our remote management program at MGB.^4^, ^9^ Compared to program entry we showed a mean home BP reduction of 14/6 mmHg (*p* < .001 for both systolic and diastolic). No serious adverse program‐related outcomes occurred.^9^ In this report we provide our hypertension remote management algorithm.

## 2 METHODS

**Figure 1:**
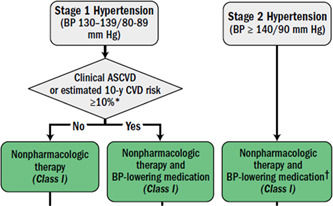
Guideline drected BP goals^2^

**Figure 2:**
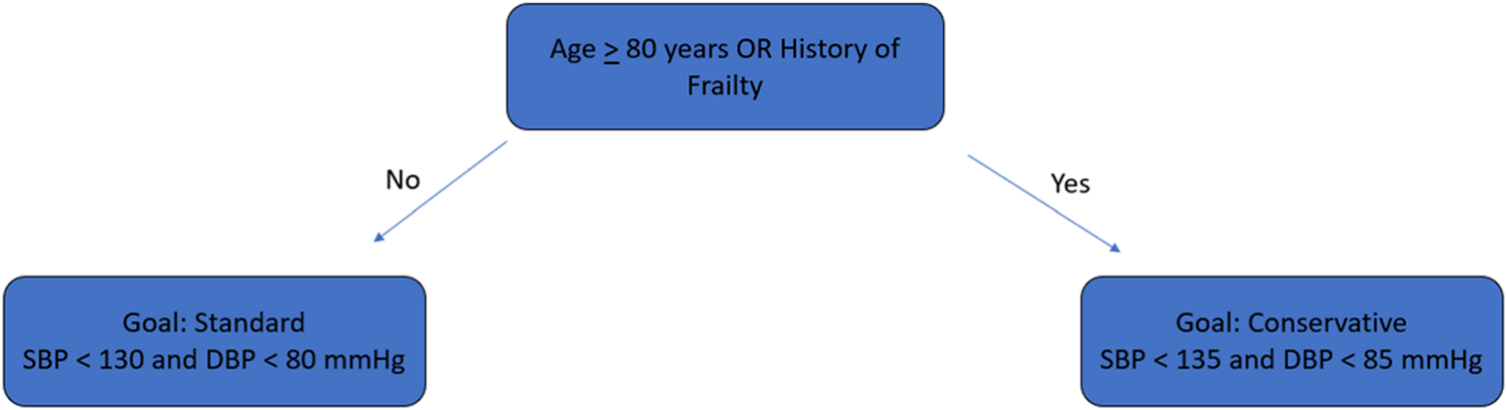
Program blood pressure goals. (Frailty is designated by use of a assistive device, cane, or walker or confirmed falls in the last 12 months, not including mechanical falls. Patients with the higher goal were given the conservative dosing approach (Appendix)).

**Figure 3:**
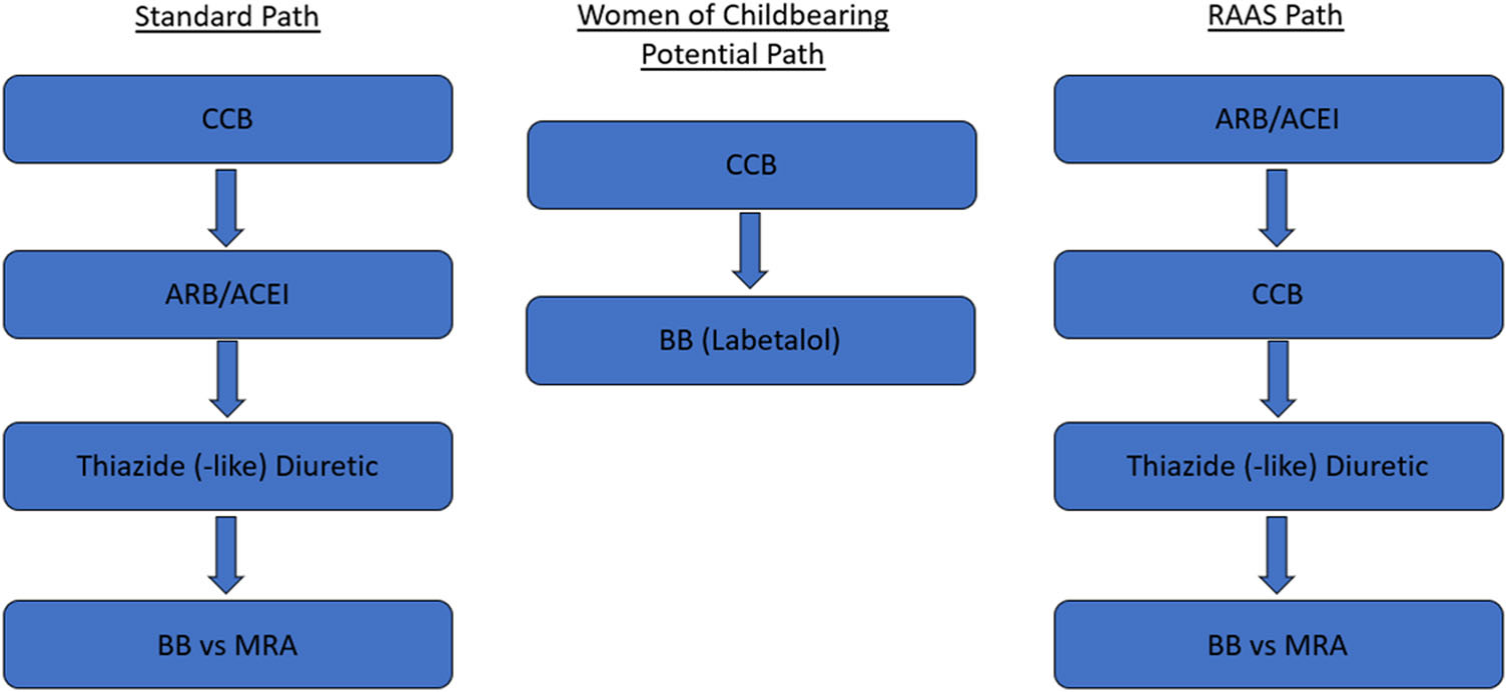
Program treatment pathways

We based our custom hypertension algorithm upon the 2017 AHA/ACC hypertension guidelines (Figure 1), with input from NICE guidance.^2^, ^10^ The algorithm uses patient‐specific characteristics to optimize a medication regimen, giving preference to calcium channel blockers (CCBs), angiotensin receptor blockers and angiotensin‐converting enzyme inhibitors (ARBs/ACEIs), and thiazide‐type diuretics (TDs), followed by mineralocorticoid receptor antagonists (MRAs) and beta blockers (BBs). Patients with baseline BP above a predefined goal—SBP < 130 and DBP < 80 mmHg (SBP < 135 and DBP < 85 mmHg for those categorized as frail (Figure 2)—and with Type 1 or 2 diabetes mellitus (DM), eGFR < 60 ml/min/1.73 m^2^ or urine microalbumin/creatinine ≥30 mg/g Cr receive renin–angiotensin–aldosterone system (RAAS) blockers (ARBs or ACEIs) first, followed by CCBs (Section Methods, 3).^11^ All other patients are started on CCBs first unless contraindicated, followed by ARBs. TDs are the typical third medication unless contraindicated (e.g., hyponatremia). MRA or BB is utilized as a fourth‐line option as appropriate (Figure 3). Combination pills are prescribed whenever possible, after doses are stabilized.

Patients with uncontrolled hypertension are identified through provider referrals and electronic health record screening aided by population health managers within the MGB health system. Non‐licensed patient navigators consent and enroll patients by telephone, mail them validated cellular or Bluetooth‐enabled BP devices, and educate them in monitoring and transmitting scheduled home BP readings (Section 3). Navigators are supervised by pharmacists, nurse practitioners, and physicians. The care team utilizes a customer relationship management software that aids workflow by employing automation to enhance communication and task generation.^12^

Home BP is monitored and averaged weekly. Transmitted BPs can be viewed in real time but most commonly are reviewed once analyzed and incorporated into weekly reports. BPs that cross preset safety thresholds are shared via alerts to clinicians. An algorithmic approach is also applied for ordering labs which are regularly monitored, with reports automatically alerting providers of critical values. Upon review of necessary clinical data, the pharmacist, under the CDTM agreement, makes medication changes via electronic prescribing.

The algorithm began as a simple text document and evolved into a digital clinical decision support tool. In its most recent iterations, all algorithmic components are digitally incorporated and operations are enhanced. All interactions with patients are documented by the navigator and reviewed by the pharmacist or nurse practitioner; a note is then routed to the patient's corresponding primary care provider and/or referring physician.

During the COVID‐19 pandemic we followed a risk mitigating strategy, prioritizing antihypertensive agents for which monitoring blood work is not required. This allowed us to treat patients who were unable to visit a laboratory due to travel restrictions. CCBs rose to the top of the prescription order, followed by BBs when reasonable. When necessary to use agents requiring labs (ARBs, ACEIs, TDs, MRAs), we used a conservative dosing strategy (Appendix).

## 3 RESULTS

1. Eligibility
  a. Patients from Massachusetts with at least one office visit to an MGB provider within the prior 3 years may be identified via the electronic medical record, or referred by their primary care provider (PCP), specialist or a population health liaison to the MGB Remote Cardiovascular (CV) Health hypertension program
    i. BP Inclusion Criteria
      1. Most recent office SBP ≥ 135 or DBP ≥ 85 mmHg, within the past 6 months (regular clinic visits only) or
      2. Average of the three most recent SBPs in the last 18 months ≥135 or DBPs ≥85 mmHg (regular clinic visits only)
      3. Exclusion Criteria
        a. age < 26 *(age* < *40 for DM)* or > 80
        b. pregnant or breastfeeding
        c. heart failure with reduced ejection fraction
        d. severe aortic stenosis
        e. bilateral renal artery stenosis
        f. eGFR < 30 ml/min/1.73m^2^
        g. orthostatic hypotension (OH)
          i. orthostatic hypotension on EHR problem list
          ii. patients reporting lightheadedness were called for a real‐time postural BP evaluation. If BP upon standing from seated position dropped by 20 mmHg systolic or 10 mmHg diastolic, they were considered to have OH
        h. terminal medical condition
        i. inability to consent
        j. weight: women > 270 lb, men > 290 lb (BP cuff unable to fit)
        k. 24‐h BP monitor within the past year demonstrating SBP < 130 and DBP < 80 mmHg
2. Treatment
  a. Treatment goals based on MGB Remote CV Health program, developed from 2017 ACC/AHA guidelines
  b. Nonpharmacologic Interventions:
    i. Education about nonpharmacological interventions is emphasized, focusing on weight loss, DASH (Dietary Approaches to Stop Hypertension) diet, dietary sodium and alcohol restriction, potassium‐rich diet, and increased physical activity. These modifications are continually reinforced with text, videos, and phone calls
    ii. For patients who are medication naïve and have SBP 130–139 or DBP 80‐89 mmHg and a 10‐year atherosclerotic cardiovascular disease (ASCVD) risk score <10%, a 2‐month trial of lifestyle modifications is initiated before pharmacologic therapy
  c. Drug Class Selection:
    i. First line—Standard Pathway
      i. All men with eGFR > 60 ml/min/1.73 m^2^ and without DM and with urine microalbumin/creatinine < 30 mg/g creatinine (Cr); all women > 45 years of age with eGFR > 60 and without DM and with urine microalbumin/creatinine < 30 mg/g Cr, and all women of childbearing potential (<45 years of age and without contraception) receive dihydropyridine CCB
        a. If CCB is not suitable due to intolerance or maximal dose already achieved, consider ARB/ACEI as next step
        b. Further information for Women of Childbearing Potential (Section 2.c.v.)
        c. During the COVID‐19 pandemic a risk mitigation strategy was employed. For anyone whose next agent would require labs as next step but who could not get labs due to the pandemic:
          i. With SBP ≥ 140 or DBP ≥ 90 mmHg treated with Standard Pathway, preference first to CCB and then to labetalol
          ii. With SBP < 140 and DBP < 90 mmHg, lifestyle modifications with 2‐month follow‐up to reassess BP and next steps as above
    ii. First line—RAAS Blockade Pathway
      i. All men and all women not deemed of childbearing potential with eGFR < 60 ml/min/1.73 m^2^ or with DM or urine microalbumin/creatinine ≥ 30 mg/g Cr receive ARB or ACEI
        a. If new start and not on ARB or ACEI, prescribe ARB; excellent safety profiles and to avoid cough^11^
        b. If already on ACEI other than lisinopril or on losartan, switch to a more potent ARB^13^, ^14^; if on lisinopril, titrate per protocol to BP goal
        c. More potent ARB^13^, ^14^ (avoid losartan) and lisinopril (or equivalent once‐daily ACEI) preferred
          i. Establish one or two ARBs as program defaults; irbesartan, valsartan used locally
        d. If ARB/ACEI is not suitable due to intolerance or maximal dose already achieved, consider CCB as next step
        e. Patients started on ARB/ACEI during COVID‐19 pandemic with limited access to labs were dosed conservatively (Appendix)
    iii. Second line
      i. ARB/ACEI or CCB, dependent on patient‐specific variables that decide the patient's treatment pathway
      ii. After reaching maximum dose of first agent, second line agent should be added if BP not at goal
        a. Maximize each dose before adding additional agent
    iv. Third line—Thiazide Type Diuretics (TD)
      i. After completing first and second line indicated pathways (RAAS inhibition and Standard), all men and all women not of childbearing potential receive TD unless contraindicated (e.g., hyponatremia)
      ii. Switch to chlorthalidone (regardless of age and frailty) if patient's systolic or diastolic BP is <10 mmHg from goal & hydrochlorothiazide is at maximum dose (Appendix)
      iii. At steady doses, TDs combined with ARB/ACEI to aid adherence
    v. Women of Childbearing Potential Pathway
      i. Women < 45 years of age and without contraception
        a. Start CCB; if at max dose of CCB and BP above goal, initiate BB; labetalol is drug of choice
    vi. Uncontrolled on maximal conventional 3‐drug regimen
      i. Order labs to screen for secondary causes of hypertension and guide treatment
        a. plasma renin activity
        b. aldosterone concentration
        c. serum potassium
        d. plasma free metanephrines
      ii. If screen for secondary causes of hypertension is positive after review by clinician lead, refer patient to hypertension specialist
      iii. If screen for secondary causes is reviewed and negative, evaluate candidacy for MRA vs BB
        a. Prescribe spironolactone or eplerenone (males) if electrolytes permit (Section 6)
        b. OR prescribe labetalol or metoprolol (if selective beta blocker preferred for other disease states: reactive airway, e.g.); heart rate must be ≥55 beats per minute (bpm)
      iv. Patients determined to have resistant hypertension, an identified secondary cause, or who have either maximized or cannot tolerate additional agents, are referred to hypertension specialist
  d. Dosing Algorithm (Appendix)
    i. Alternative agents within the same class may be used instead of a preferred agent if a contraindication exists, if the patient is stable on another appropriate agent, or for cost considerations
  e. Medication Management
    i. Details of algorithmic starting doses, intervals and rules regarding when to titrate are below
3. BP Monitoring
  a. An instructional pamphlet is included with each BP cuff sent to patients, to educate them on proper technique. Program staff are trained and available to answer questions
  b. Home BP monitoring (HBPM) is performed upon program enrollment and after each change in BP medication regimen:
    i. BP is recorded twice daily—morning and evening—always before medications
    ii. Patients measure BP in duplicate each time, with two consecutive seated readings taken 1–2 min apart while at rest
    iii. BP recordings continue for 7 days/28 readings (optimal), 3 days/12 readings (minimal)
    iv. Weekly average of all measurements is used to confirm diagnosis of hypertension or to assess hypertension control. Outlier BP values (>3 SD from the mean) are removed from calculation. If HBPM average SBP ≥ 130 or DBP ≥ 80 mmHg, diagnosis of hypertension is confirmed
  c. If patient's baseline average home weekly SBP < 130 and DBP < 80 mmHg (or SBP < 135 and DBP < 85 mmHg with frailty) after enrollment but before any medication changes, patient is diagnosed with white coat hypertension or white coat effect and enters maintenance
  d. If patient's average weekly SBP < 130 and DBP < 80 mmHg (or SBP < 135 and DBP < 85 mmHg with frailty) 2 weeks after medication titration, patient's HTN is considered controlled and patient enters maintenance, pending labs as appropriate
  e. If patient's average weekly SBP ≥ 130 or DBP ≥ 80 mmHg (or SBP ≥ 135 or DBP ≥ 85 mmHg with frailty), then escalate dose or add additional agent
    i. If either SBP or DBP dictates patient is above goal but SBP < 105 mmHg, DBP < 50 mmHg, or HR < 55 bpm for BB patients, a conversation with physician clinical lead to decide next steps is triggered
    ii. Do not increase BB dose if HR < 55 bpm
  f. Continue to titrate and check BP. Patients should be on a new dose of medication for at least 1 week, at least 6 weeks for MRA, before rechecking BP for next titration. Once patient's average weekly SBP < 130 and DBP < 80 mmHg (or SBP < 135 and DBP < 85 mmHg with frailty), patient may enter maintenance, pending labs as appropriate
4. Critical Blood Pressure Values
  a. An alert report is generated daily and triaged by the pharmacist or nurse practitioner, with phone calls to the patient to assess for alarm symptoms (Section 5), proper measurement technique and medication adherence for patients meeting these criteria:
    i. If on any ONE day:
      i. SBP ≤ 90 mmHg or
      ii. DBP ≥ 120 mmHg or
      iii. SBP ≥ 200 mmHg
    ii. OR if within a 7‐day period
      i. 2 days of SBP ≥ 190 mmHg
    iii. OR if within a 7‐day period
      i. 3 days of SBP ≥ 180 mmHg
  b. A pamphlet included with each BP cuff explains and provides direction for potentially dangerous BP readings
    i. A patient with SBP ≥ 200 mmHg but without alarm symptoms (Section 5) is instructed to recheck BP in 1 h, ensuring proper technique
    ii. A patient with a SBP ≤ 90 mmHg with dizziness or lightheadedness is instructed to recheck BP in 1 h, ensuring proper technique
      i. If alerting BP levels or symptoms persist, patients are to call the program immediately; off hours, they are to call their PCP
      ii. Thresholds were selected to provide simple patient safety materials and guidance
5. Alarm Symptoms
  a. A pamphlet included with each BP cuff explains potentially dangerous symptoms of high and low BP, and possible adverse reactions to medication. If experiencing the following symptoms at home, they are instructed to call emergency medical services (EMS). If reporting any of these symptoms to our program, they are instructed to call EMS and immediate notification of program clinical leads and pharmacist is prompted:
    i. chest pain
    ii. acute shortness of breath
    iii. severe headache
    iv. throat, tongue, lip or mouth swelling
    v. unclear thinking
    vi. visual changes
    vii. nausea/vomiting
6. Lab Monitoring
  a. General program enrollment labs required
    i. baseline BMP within 12 months of enrollment
    ii. lipid panel within the past 3 years for ASCVD risk assessment
  b. Program level warnings automatically generated for the pharmacist:
    i. Recommendation for ACEI/ARB and most recent eGFR <  60 ml/min/1.73 m^2^
    ii. Recommendation for ACEI/ARB and most recent K > 4.5 mmol/L
    iii. Recommendation for TD and most recent Na < 140 mmol/L
    iv. Recommendation for TD and most recent K < 4.2 mmol/L
    v. Recommendation for ACEI/ARB/TD and increase in last K > 15% from baseline (based on enrollment in the program) AND most recent K > 5 mmol/L
      i. Of note, rules surrounding K, Na, eGFR and titration are observed and acknowledged, or plan is altered and documented
  c. Specific Drug Classes
    i. CCB Labs
      i. General program enrollment labs only; no specific labs needed to start or titrate CCB
    ii. ARB/ACEI Labs
      i. General program enrollment labs needed to start or titrate ARB/ACEI. Also required:
        a. Most recent K < 5.2 mmol/L
        b. Most recent eGFR ≥ 30 ml/min/1.73m2
        c. Most recent eGFR < 30% decrease from baseline
        d. BMP drawn 1‐2 weeks after initiation of ARB/ACEI and after the last titration. If there is a potential for three or more titrations for one medication (i.e., irbesartan 75, 150, 300 mg), labs can be checked every other titration if previous values are within normal limits
        e. Repeat labs should be obtained if there was an increase in creatinine ≥ 30% from baseline or an increase in K ≥ 15% from baseline AND K > 5 mmol/L, or down‐titrate previous associated step in regimen escalation and recheck labs
        f. Labs must be obtained when final dosing is reached; these can be drawn within 4 weeks of last dose change
    iii. TD Labs
      i. General program enrollment labs needed to start or titrate TD. Also required:
        a. Most recent K ≥ 3.5 mmol/L
        b. Most recent Na ≥ 135 mmol/L
        c. Most recent eGFR ≥ 30 ml/min/1.73 m^2^
        d. BMP should be drawn 1–2 weeks after initiation of TD and after the last titration. If there is a potential for three or more titrations for one medication (i.e., HCTZ 12.5 mg daily, HCTZ 25 mg daily, chlorthalidone 25 mg daily), labs can be checked every other titration if previous values are within normal limits
        e. In this sequence chlorthalidone is considered as the up‐titration of TD (Appendix)
        f. Repeat labs should be obtained if there was an increase in creatinine ≥ 30% from baseline, a decrease in K < 3.5 mmol/L or Na < 135 mmol/L from baseline, or down‐titrate previous associated step in regimen escalation and recheck labs
        g. Labs must be obtained when final dosing is reached; these can be drawn within 4 weeks of last dose change
    iv. MRA Labs
      i. General program enrollment labs needed to start or titrate MRA. Also required:
        a. Titrations separated 6 weeks apart to allow for maximal BP reduction from dose change
        b. Most recent K < 5.2 mmol/L (if K > 4.5 mmol/L start conservative dose, discuss all labs with HTN clinical lead; Appendix)
        c. Most recent Na ≥ 135 mmol/L
        d. Most recent eGFR ≥ 30 ml/min/1.73 m^2^
        e. Most recent eGFR < 30% decrease from baseline
        f. BMP should be drawn 1–2 weeks after initiation of MRA and after the last titration. If there is a potential for three or more titrations for one medication (e.g., spironolactone 12.5 mg daily, 25 mg daily, 50 mg daily) labs can be checked every other titration, if previous values are within normal limits
        g. Repeat labs should be obtained if there was an increase in creatinine ≥ 30% from baseline, an increase in K ≥ 15% from baseline AND K > 5 mmol/L, or Na < 135 mmol/L from baseline, or down‐titrate previous associated step in regimen escalation and recheck labs
        h. Labs must be obtained when final dosing is reached; these can be drawn within 4 weeks of last dose change
    v. BB Labs
      i. General program enrollment labs only; no specific drug class labs needed to start or titrate BB

## 4 DISCUSSION

We describe the detailed methods of our remote HTN management program that uses an algorithmic approach to control BP. The program leverages non‐licensed patient navigators, pharmacists with CDTM agreements, nurse practitioner and physician support, customer relationship management software, and cellular and Bluetooth‐enable devices with data integration. Goals of maximizing efficiency, streamlining and innovating processes have been central to program development. The results yield an iteratively developed and real‐life tested hypertension algorithm that has proven effective for hypertension management.^9^, ^12^

For patients with chronic diseases such as hypertension, remote monitoring and application of CDTM pharmacy agreements can increase patients' disease‐specific knowledge, improve self‐management and shared decision‐making, and provide the necessary prescriptions and follow‐up.^7^, ^15^ The Federal Food, Drug and Cosmetic Act of 1938 and subsequent Durham‐Humphrey amendment in 1951 led to the legal separation of prescribing—to be done by physicians—and dispensing, to be done by pharmacists, and to the designation of prescription versus non‐prescription medications.^16^ Before this, pharmacists could prescribe medications legally. An early model for CDTM was put forth by the Indian Health Services (IHS) in the 1960s when pharmacists in this organization began assuming an active role in medication management.^16^ By the 1970s the IHS had received federal funding for the Pharmacist Practitioner Program which saw pharmacists provide drug therapy management in collaboration with physicians.^17^, ^18^ The program was well received by both physicians and patients.^19^ In the 1980s a study showed that clinical pharmacists using physician‐supervised protocols managed patients in a skilled nursing facility with strong clinical results and cost savings compared to those without pharmacist involvement.^20^ In 1995 the Veterans Health Administration began allowing pharmacists to practice CDTM.^21^ In 1997, 2003, and again in 2015 the American College of Clinical Pharmacy released position statements encouraging and providing guidance surrounding CDTM and the pharmacist role.^16^, ^21^, ^22^ Now in the 2020s nearly every state in the country allows for pharmacists to practice CDTM, with roles and responsibilities legislated by state.^22^ While this program focuses on hypertension management, CDTM is employed across many chronic disease states including dyslipidemia, diabetes, anticoagulation, chronic pain, outpatient antimicrobial infusion protocols, and others.^23^

Certain key differences distinguish our remote hypertension management program from others being trialed and implemented around the country. Historically, CDTM pharmacy was practiced in the clinic setting.^21^ Innovative hypertension programs focus on telehealth and remote management. In many remote management programs, the pharmacist calls the patient and discusses lifestyle modifications and medication adherence and makes medication changes.^6^, ^7^, ^24^, ^25^ Studies demonstrate the positive impact pharmacists can have on improving BP control.^24^, ^25^ Utilizing patient navigators, our program allows pharmacists to task shift patient calls and ancillary duties and to dedicate more time to reviewing clinical data, making clinical decisions and prescribing medications.^9^

Remote management provides flexibility for patients and providers, avoiding the constraints of appointment windows and travel to office visits. Utilizing CDTM and pharmacists, physicians gain time to spend with complex patients. Having an entirely remote program and using customer relationship management software with our integrated algorithm has also allowed for adaptability, which has been essential during the pandemic. Our algorithmic approach was changed in real time in response to the emergency. In addition, managing patients remotely using an omni‐channel approach to communication has been effective. Phone calls, texting, electronic health record communication, and e‐mails were all incorporated after receiving consent. Information from patients and their electronic health record is imported into our digital clinical decision support tool, rendering a customizable algorithmic treatment plan that can be facilitated by patient navigators. This completes the process of placing remote monitoring within a full service, CDTM‐based, algorithmically and digitally supported hypertension program.

### 4.1 LIMITATIONS

Despite prescribing guideline‐recommended, well‐tolerated, low‐cost generic BP agents, patients may have clinical contraindications. Cost can be limiting for some. Prioritization logic is built to mitigate roadblocks, but exceptions to the rule exist and cannot always be accounted for. In these instances, individual deviations from the medication algorithm must be discussed and executed. With a finite number of treatment options presented, some patients fail to reach goal after all available algorithmic regimens are exhausted. Several agents used require blood tests to maintain clinical safety; access to laboratory testing is limited for some.

Home BP devices and data transmission are essential to program operations, but BP cuff utilization is difficult for some patients. We mitigate technical device issues with text instructions, demonstration videos, navigator device set‐up calls, and continued coaching and troubleshooting throughout a patient's program journey. Issues with data transmission are handled by phone calls; if automated BPs are not transmitted, manual reporting and averaging of BPs by patient navigators occurs.

Patient navigators lack the background education and training of medical professionals. Clinical concerns must be relayed to licensed providers and triaged. Communication is swift but not immediate for patients. Cost considerations must be factored when implementing a program with physicians, nurse practitioner, pharmacists, patient navigators, as well as data and IT support.

Our algorithm is purposefully dynamic and iterative; a one‐time snapshot cannot capture it in perpetuity. In addition, regular reassessments for safety and efficacy are necessary.

Remote hypertension management has been successful for many but not all, due partly to difficulty connecting virtually, barriers with technology, and engaging with the remote model.^9^, ^15^

### 4.2 CONCLUSION

We have shown that an algorithmic approach in a digital remote hypertension program has been successful in managing hypertension.^9^ A strong focus on algorithmic decision‐making, streamlining of tasks and responsibilities, and utilization of a CDTM agreement help make this scalable, with the potential to reach large populations of patients who need better hypertension control.

## Data Availability

Data sharing not applicable to this article as no datasets were generated or analyzed during the current study.

## 1

Table A1, A2, A3, A4, A5, A6, A7, A8, A9, A10, A11, A12

**Table A1:** ACE inhibitor recommendation

	Lisinopril (Zestril, Prinvil)	Benazepril (Lotensin)	Captopril (Capoten)	Enalapril (Vasotec)	Quinapril (Accupril)	Ramipril (Altace)	Trandolapril (Mavik)	Perindopril (Aceon)	Fosinopril (Monopril)	Moexipril (Univasc)
Initial dose	**10 mg daily**	10 mg BID	25 mg TID	5 mg BID	10 mg BID	2.5 mg BID	1 mg BID (2 mg daily in black patients)	4 mg BID	10 mg BID	7.5 mg BID
Titration	**10 mg daily** ↓ **20 mg daily** ↓ **40 mg daily**	10 mg BID ↓ Irbesartan 75 mg daily ↓ Irbesartan 150 mg daily ↓ Irbesartan 300 mg daily	25 mg TID ↓ Irbesartan 150 mg daily ↓ Irbesartan 300 mg daily	5 mg BID ↓ Irbesartan 75 mg daily ↓ Irbesartan 150 mg daily ↓ Irbesartan 300 mg daily	10 mg BID ↓ Irbesartan 75 mg daily ↓ Irbesartan 150 mg daily ↓ Irbesartan 300 mg daily	2.5 mg BID ↓ Irbesartan 75 mg daily ↓ Irbesartan 150 mg daily ↓ Irbesartan 300 mg daily	1 mg BID ↓ Irbesartan 75 mg daily ↓ Irbesartan 150 mg daily ↓ Irbesartan 300 mg daily	4 mg BID ↓ Irbesartan 150 mg daily ↓ Irbesartan 300 mg daily	10 mg BID ↓ Irbesartan 150 mg daily ↓ Irbesartan 300 mg daily	7.5 mg BID ↓ Irbesartan 150 mg daily ↓ Irbesartan 300 mg daily
Target dose	**40 mg daily**	Irbesartan 300 mg daily	Irbesartan 300 mg daily	Irbesartan 300 mg daily	Irbesartan 300 mg daily	Irbesartan 300 mg daily	Irbesartan 300 mg daily	Irbesartan 300 mg daily	Irbesartan 300 mg daily	Irbesartan 300 mg daily
Max dose	**40 mg daily**	Irbesartan 300 mg daily	Irbesartan 300 mg daily	Irbesartan 300 mg daily	Irbesartan 300 mg daily	Irbesartan 300 mg daily	Irbesartan 300 mg daily	Irbesartan 300 mg daily	Irbesartan 300 mg daily	Irbesartan 300 mg daily
Monitoring		BP, heart rate, BUN, SCr, K per above protocol								
Contraindications		Allergy, angioedema, intolerable cough, moderate to severe aortic stenosis, bilateral renal artery stenosis, pregnancy coadministration with aliskiren in patients with diabetes								

*Note*: Bold text represents preferred medications.

**Table A2:** ACE inhibitor conservative dosing recommendation

	Lisinopril (Zestril, Prinvil)	Benazepril (Lotensin)	Captopril (Capoten)	Enalapril (Vasotec)	Quinapril (Accupril)	Ramipril (Altace)	Trandolapril (Mavik)	Perindopril (Aceon)	Fosinopril (Monopril)	Moexipril (Univasc)
Initial Dose	**5 mg daily**	5 mg BID	12.5 mg TID	2.5 mg BID	10 mg BID	1.25 mg BID	1 mg BID	2 mg BID	5 mg BID	3.75 mg BID
Titration	**5 mg daily** ↓ **10 mg daily** ↓ **20 mg daily** ↓ **30 mg daily** ↓ **40 mg daily**	5 mg BID ↓ Irbesartan 75 mg daily ↓ Irbesartan 150 mg daily ↓ Irbesartan 150 mg daily ↓ Irbesartan 300 mg daily	12.5 mg TID ↓ Irbesartan 75 mg daily ↓ Irbesartan 150 mg daily ↓ Irbesartan 300 mg daily	2.5 mg BID ↓ Irbesartan 75 mg daily ↓ Irbesartan 150 mg daily ↓ Irbesartan 150 mg daily ↓ Irbesartan 300 mg daily	10 mg BID ↓ Irbesartan 75 mg daily ↓ Irbesartan 150 mg daily ↓ Irbesartan 300 mg daily	1.25 mg BID ↓ Irbesartan 75 mg daily ↓ Irbesartan 150 mg daily ↓ Irbesartan 150 mg daily ↓ Irbesartan 300 mg daily	1 mg BID ↓ Irbesartan 75 mg daily ↓ Irbesartan 150 mg daily ↓ Irbesartan 300 mg daily	2 mg BID ↓ Irbesartan 75 mg daily ↓ Irbesartan 150 mg daily ↓ Irbesartan 300 mg daily	5 mg BID ↓ Irbesartan 75 mg daily ↓ Irbesartan 150 mg daily ↓ Irbesartan 300 mg daily	3.75 mg BID ↓ Irbesartan 75 mg daily ↓ Irbesartan 150 mg daily ↓ Irbesartan 300 mg daily
Target dose	**40 mg daily**	Irbesartan 300 mg daily	Irbesartan 300 mg daily	Irbesartan 300 mg daily	Irbesartan 300 mg daily	Irbesartan 300 mg daily	Irbesartan 300 mg daily	Irbesartan 300 mg daily	Irbesartan 300 mg daily	Irbesartan 300 mg daily
Max dose	**40 mg daily**	Irbesartan 300 mg daily	Irbesartan 300 mg daily	Irbesartan 300 mg daily	Irbesartan 300 mg daily	Irbesartan 300 mg daily	Irbesartan 300 mg daily	Irbesartan 300 mg daily	Irbesartan 300 mg daily	Irbesartan 300 mg daily

*Note*: For patients on a diuretic of >25 mg thiazide diuretic equivalent dose, use the conservative dosing path. For patients who have met criteria for conservative goal <135/85 mmHg, use conservative dosing path, Bold text represents preferred medications.

**Table A3:** ARB recommendation

	Losartan (Cozaar)	Candesartan (Atacand)	Valsartan (Diovan)	Irbesartan (Avapro)	Olmesartan (Benicar)	Telmisartan (Micardis)	Azilsartan (Edarbi)	Eprosartan (Teveten)
Initial dose	50 mg daily	16 mg daily	80 mg daily	**150 mg daily**	20 mg daily	40 mg daily	80 mg daily	600 mg daily
Titration	50 mg daily ↓ Irbesartan 150 mg daily ↓ Irbesartan 300 mg daily	16 mg daily ↓ 32 mg daily	80 mg daily ↓ 160 mg daily ↓ 240 mg daily ↓ 320 mg daily	**150 mg daily** ↓ **300 mg daily**	20 mg daily ↓ 40 mg daily	40 mg daily ↓ 80 mg daily	80 mg daily	600 mg daily ↓ 800 mg daily
Target dose	Irbesartan 300 mg daily	12– 32 mg daily	160–320 mg daily	**300 mg daily**	40 mg daily	40‐80 mg daily	80 mg daily	600‐ 800 mg daily
Max dose	Irbesartan 300 mg daily	32 mg daily	320 mg daily	**300 mg daily**	40 mg daily	80 mg daily	80 mg daily	800 mg daily
Monitoring		BP, heart rate, BUN, SCr, K per above protocol						
Contraindications		Allergy, angioedema, moderate to severe aortic stenosis, bilateral renal artery stenosis, pregnancy, coadministration with aliskiren in patients with diabetes						

*Note*: Bold text represents preferred medications

**Table A4:** ARB conservative dosing recommendation

	Losartan (Cozaar)	Candesartan (Atacand)	Valsartan (Diovan)	Irbesartan (Avapro)	Olmesartan (Benicar)	Telmisartan (Micardis)	Azilsartan (Edarbi)	Eprosartan (Teveten)
**Initial Dose**	25 mg daily	8 mg daily	40 mg daily	**75 mg daily**	20 mg daily	20 mg daily	40 mg daily	400 mg daily
**Titration**	25 mg daily ↓ Irbesartan 75 mg daily ↓ Irbesartan 150 mg daily ↓ Irbesartan 300 mg daily	8 mg daily ↓ 16 mg daily ↓ 32 mg daily	40 mg daily ↓ 80 mg daily ↓ 160 mg daily ↓ 240 mg daily ↓ 320 mg daily	**75 mg daily** ↓ **150 mg daily** ↓ **300 mg daily**	20 mg daily ↓ 40 mg daily	20 mg daily ↓ 40 mg daily ↓ 80 mg daily	40 mg daily ↓ 80 mg daily	400 mg daily ↓ 600 mg daily ↓ 800 mg daily
**Target dose**	Irbesartan 300 mg daily	12–32 mg daily	160–320 mg daily	**300 mg daily**	40 mg daily	40‐80 mg daily	80 mg daily	600‐ 800 mg daily
**Max dose**	Irbesartan 300 mg daily	32 mg daily	320 mg daily	**300 mg daily**	40 mg daily	80 mg daily	80 mg daily	800 mg daily

*Note*: For patients on a diuretic of >25 mg thiazide diuretic equivalent dose, use the conservative dosing path. For patients who have met criteria for conservative goal <135/85 mmHg, use conservative dosing path, Bold text represents preferred medications.

**Table A5:** Calcium channel blocker recommendation: **Dihydropyridines**

	Amlodipine (Norvasc)	Felodipine (Plendil)	Nifedipine ER (Nifedical XL, Procardia XL)
Initial dose	**5 mg daily**	5 mg daily	30 mg daily
Titration	**5 mg daily** ↓ **10 mg daily**	5 mg daily ↓ 10 mg daily	30 mg daily ↓ 60 mg daily ↓ 90 mg daily ↓ 120 mg daily
Max dose	**10 mg daily**	10 mg daily	120 mg daily
Monitoring	Heart rate, BP, signs of edema		
Contraindications	Hypersensitivity to any component of the drug		

*Note*: Bold text represents preferred medications.

**Table A6:** Dihydropyridines conservative dosing

	Amlodipine (Norvasc)	Felodipine (Plendil)	Nifedipine ER (Nifedical XL, Procardia XL)
Initial dose	**2.5 mg daily**	2.5 mg daily	30 mg daily
Titration	**2.5 mg daily** ↓ **5 mg daily** ↓ **10 mg daily**	2.5 mg daily ↓ 5 mg daily ↓ 10 mg daily	30 mg daily ↓ 60 mg daily ↓ 90 mg daily ↓ 120 mg daily
Max dose	**10 mg daily**	10 mg daily	120 mg daily

*Note*: For patients who have met criteria for conservative goal <135/85 mmHg, use conservative dosing path. Bold text represents preferred medications.

**Table A7:** MRA recommendation

	Spironolactone (Aldactone)	Eplerenone (Inspra)
Initial dose	**25 mg daily**	50 mg daily
Titration	**50 mg daily**	50 mg twice daily
Target dose	**Spironolactone 25 mg daily**	Eplerenone 50 mg daily
Max dose	**Spironolactone 50 mg daily**	Eplerenone 50 mg twice daily
Monitoring		BP, heart rate, BUN, SCr, K per above protocol
Contraindications		Allergy, moderate to severe aortic stenosis, bilateral renal artery stenosis, pregnancy, gynecomastia (Men start with Eplerenone)*
		Use conservative path for K > 4.5 mmol/L

*Note*: Male patients should be started on Eplerenone rather than Spironolactone when possible. Bold text represents preferred medications.

**Table A8:** MRA conservative recommendation

	Spironolactone (Aldactone)	Eplerenone (Inspra)*
Initial Dose	**12.5 mg daily**	50 mg daily
Titration	**12.5 mg daily** ↓ **25 mg daily** ↓ **50 mg daily**	25 mg daily ↓ 50 mg daily ↓ 50 mg twice daily
Target dose	**25 mg daily**	Ep 50 mg daily
Max dose	**50 mg daily**	Ep 50 mg twice daily

*Note*: For patients on a diuretic of >25 mg thiazide diuretic equivalent dose, use the conservative dosing path. For patients who have met criteria for conservative goal <135/85 mmHg, use the conservative dosing path. Bold text represents preferred medications.

**Table A9:** Beta blocker recommendation

	Atenolol (Tenormin)	Carvedilol (Coreg)	Carvedilol ER (Coreg CR)	Labetalol (Trandate)	Metoprolol succinate (Toprol‐XL)
Initial dose	25 mg daily	6.25 mg BID	20 mg daily	**100 mg BID**	**25 mg daily**
Titration	25 mg daily ↓ Toprol‐XL 50 mg daily ↓ Toprol‐XL 100 mg daily ↓ Toprol‐XL 150 mg daily ↓ Toprol‐XL 200 mg daily	6.25 mg BID ↓ 12.5 mg BID ↓ 25 mg BID	20 mg daily ↓ 40 mg daily ↓ 80 mg daily	**100 mg BID** ↓ **200 mg BID** ↓ **300 mg BID** ↓ **400 mg BID**	**25 mg daily** ↓ **50 mg daily** ↓ **100 mg daily** ↓ **150 mg daily** ↓ **200 mg daily**
Target dose	Toprol‐XL 100‐200 mg daily	25 mg BID	80 mg daily	**100–300mg BID**	**100–200 mg daily**
Max dose	Toprol‐XL 200 mg daily	25 mg BID	80 mg daily	**400 mg BID**	**200 mg daily**
Monitoring	BP, heart rate				
Contraindications	Hypersensitivity to any component of the drug, severe bradycardia, second/third‐degree heart block (except with artificial pacemaker), cardiogenic shock, asthma or obstructive airway disease, conditions causing severe hypotension				

*Note*: Bold text represents preferred medications.

**Table A10:** Beta blocker conservative dosing recommendation

	Atenolol (Tenormin)	Carvedilol (Coreg)	Carvedilol ER (Coreg CR)	Labetalol (Trandate)	Metoprolol succinate (Toprol‐XL)
Initial dose	12.5 mg daily	3.125 mg BID	10 mg daily	**50 mg BID**	**12.5 mg daily**
Titration	12.5 mg daily ↓ Toprol‐XL 25 mg daily ↓ Toprol‐XL 50 mg daily ↓ Toprol‐XL 100 mg daily ↓ Toprol‐XL 150 mg daily ↓ Toprol‐XL 200 mg daily	3.12 mg BID ↓ 6.25 mg BID ↓ 12.5 mg BID ↓ 25 mg BID	10 mg daily ↓ 20 mg daily ↓ 40 mg daily ↓ 80 mg daily	**50 mg BID** ↓ **100 mg BID** ↓ **200 mg BID** ↓ **300 mg BID** ↓ **400 mg BID**	**12.5 mg daily** ↓ **25 mg daily** ↓ **50 mg daily** ↓ **100 mg daily** ↓ **150 mg daily** ↓ **200 mg daily**
Target dose	Toprol‐XL 100 mg‐ 200 mg daily	25 mg BID	80 mg daily	**100–300 mg BID**	**100–200 mg daily**
Max dose	Toprol‐XL 200 mg daily	25 mg BID	80 mg daily	**400 mg BID**	**200 mg daily**

*Note*: For patients who have met criteria for conservative goal <135/85 mmHg, use the conservative dosing path. Bold text represents preferred medications.

**Table A11:** Thiazide diuretic recommendation

	Chlorthalidone (Thalitone)	Hydrochlorothiazide (Microzide)
Initial dose	25 mg daily	**12.5 mg daily**
Titration	25 mg daily	**12.5 mg daily**
		**↓**
		**25 mg daily**
Target dose	25 mg daily	**25 mg daily**
Max dose	25 mg daily	**25 mg daily**
Monitoring	Weight, BP, serum electrolytes, kidney function	
Contraindications	Hypersensitivity to the drug or sulfonamide‐derived drugs, history of gout, known anuria	

*Note*: Bold text represents preferred medications.

**Table A12:** Thiazide diuretic conservative dosing recommendation

	Chlorthalidone (Thalitone)	Hydrochlorothiazide (Microzide)
**Initial dose**	12.5 mg	**12.5 mg daily**
**Titration**	12.5 mg daily	**12.5 mg daily**
	↓	↓
	25 mg daily	**25 mg daily**
**Target dose**	25 mg daily	**25 mg daily**
**Max dose**	25 mg daily	**25 mg daily**

*Note*: For patients who have met criteria for conservative goal <135/85 mmHg, use conservative dosing path. Bold text represents preferred medications.
